# Genetic Basis of Dorper Sheep (*Ovis aries*) Revealed by Long-Read *De Novo* Genome Assembly

**DOI:** 10.3389/fgene.2022.846449

**Published:** 2022-04-11

**Authors:** Guoyan Qiao, Pan Xu, Tingting Guo, Yi Wu, Xiaofang Lu, Qingfeng Zhang, Xue He, Shaohua Zhu, Hongchang Zhao, Zhihui Lei, Weibo Sun, Bohui Yang, Yaojing Yue

**Affiliations:** ^1^ Lanzhou Institute of Husbandry and Pharmaceutical Sciences of Chinese Academy of Agricultural Sciences, Lanzhou, China; ^2^ State Key Laboratory of Grassland Agro-Ecosystems, Ministry of Agriculture and Rural Affairs, Engineering Research Center of Grassland Industry, Ministry of Education, College of Pastoral Agriculture Science and Technology, Lanzhou University, Lanzhou, China; ^3^ Key Laboratory of Grassland Livestock Industry Innovation, Ministry of Agriculture and Rural Affairs, Engineering Research Center of Grassland Industry, Ministry of Education, College of Pastoral Agriculture Science and Technology, Lanzhou University, Lanzhou, China; ^4^ Tianjin Aoqun Animal Husbandry Pty., Ltd., Tianjin, China; ^5^ The Enterprises Key Laboratory of Tianjin Meat-Type-Sheep Genetics and Breeding, Tianjin, China

**Keywords:** dorper sheep, reference genome, genetic basis, composite breed, allele-specific expression (ASE)

## Abstract

Dorper sheep (*Ovis aries*) (DPS), developed in the 1930s by crossing Dorset Horn and Blackhead Persian sheep in South Africa, is a world-famous composite breed for mutton production. The genetic basis underlying this breed is yet to be elucidated. Here, we report the sequencing and assembly of a highly contiguous Dorper sheep genome via integration of Oxford Nanopore Technology (ONT) sequencing and Hi-C (chromatin conformation capture) approaches. The assembled genome was around 2.64 Gb with a contig N50 of 73.33 Mb and 140 contigs in total. More than 99.5% of the assembled sequences could be anchored to 27 chromosomes and they were annotated with 20,450 protein-coding genes. Allele-specific expression (ASE) genes of Dorper sheep were revealed through ASE analysis and they were involved in the immune system, lipid metabolism, and environmental adaptation. A total of 5,701 and 456 allelic sites were observed in the SNP and indels loci identified from relevant whole-genome resequencing data. These allelic SNP and INDEL sites were annotated in 1,002 and 294 genes, respectively. Moreover, we calculated the number of variant sites and related genes derived from the maternal and paternal ancestors, revealing the genetic basis of outstanding phenotypic performance of Dorper sheep. In conclusion, this study reports the first reference genome of Dorper sheep and reveals its genetic basis through ASE. This study also provides a pipeline for mining genetic information of composite breeds, which has an implication for future hybrid-breeding practices.

## 1 Introduction

The sheep (*Ovis aries*) was one of the first animals domesticated for agricultural purposes (Hiendleder, Kaupe, Wassmuth, & Janke, 2002). Currently, the total number of domesticated sheep in the world exceeds one billion. They are an important source of meat, wool, and dairy products, and play an important role in the global agricultural economy. Sheep are widely distributed in the cold zone, in the tropics, and at high elevations due to their rich phenotypic variations of breeds for different production targets (Alberto et al., 2018). High quality genomes of sheep are the basis for systematically exploring their evolution and analyzing the unique biological traits, which is of great significance for the conservation and utilization of genetic resources and the mining of genetic characteristics (Roth, 2019).

The Dorper sheep is an easy-care, fast-growing, meat-producing hair sheep, that was developed and became the second largest breed in South Africa by crossing Dorset Horn with Blackhead Persian in the 1930s, then spread to many other countries throughout the world (Cloete, Snyman, & Herselman, 2000). Hair sheep are widely used to breed heat-tolerant lambs, which is particularly important given the current and future pressures on sheep breeding that are associated with climate change. Unlike wool sheep, Dorper sheep don’t require shearing, crutching, or mulesing, and they are much less prone to flystrike (Pollott, 2011). They have high fertility and maternal instinct, combined with a high growth rate and hardiness. They reputably do well in various environmental and feeding conditions, particularly intensive feeding system (Tesema et al., 2020). However, only limited genomic information is available for this important breed. With the rapid development and lowering cost of sequencing technologies, the use of genomics in mining livestock genetic diversity is becoming more widespread (van Dijk, Jaszczyszyn, Naquin, & Thermes, 2018). Up to now, the genomes of at least 15 domestic sheep breeds have been assembled (Jiang et al., 2014; Li et al., 2021; Davenport et al., 2022). There are various breeds of sheep being commercially bred, but Dorper holds significance due to its special characteristics. Therefore, an annotated complete genome is of great significance for the study of this sheep breed.

As mentioned earlier, Dorper sheep is a composite breed whose ancestors were Blackhead Persian sheep and Dorset Horn sheep. Blackhead Persian sheep locally adapted to warm and dry environments are likely to carry genes that could resist the negative effects of global warming and increased aridity. The Dorset Horn sheep is one of the best meat sheep breeds in the world and yields heavily muscled carcasses, best known for its ability to produce a lamb crop any time of the year (Porter, Alderson, Hall, & Sponenberg, 2016). In a population structure research of African sheep breeds, Dorper sheep were clustered in between the Dorset and Blackhead Persian clusters based on PCA and ADMIXTURE analysis. The research confirmed the relationship between Doper sheep and their ancestors (Dzomba et al., 2020). Given the breeding and genomic background of Doper sheep, we can determine that this breed inherits desirable traits from the Dorset Horn and Blackhead Persian sheep. The divergence of gene expression is an essential source of phenotypic diversity and the co-expression of alleles at the parent locus underpins certain traits of diploid hybrids (Knight 2004). Numerous studies have suggested that only one allele is expressed in heterozygotes, and monoallelic expression or an imbalance in heterozygote allelic expression has been studied in depth in humans and other mammals (Song et al., 2013). A study with tissues from goats’ hybrids uncovered multiple genes exhibiting allele-specific expression (Cao et al., 2019). Wang et al. investigated the global allele-specific expression and splicing across adipose and muscle from 15 adult crossbred sheep successfully identified ASE genes with a potential role in muscle growth and fat deposition (Wang et al., 2022). These studies have demonstrated that ASE (allele-specific expression) could contribute to explaining the genetic basis of composite breeds. In this study, we tried to establish a workflow to investigate the ancestral genomic components of Dorper sheep by ASE analysis.

The selection of reference genomes is also crucial to complete ASE analysis. In essence, a reference assembly is an attempt at a complete representation of the nucleotide sequence of an individual genome. This reference assembly allows for a shortcut when sequencing future samples/individuals as they can be mapped to the reference, instead of building a new assembly.

In this study, we need to compare the transcriptome data of Dorper sheep with the appropriate reference genome to detect SNP and INDEL that can represent the Population of the Dorper sheep. There are many different versions of the sheep genome, however, the genomes of different sheep breeds are not exactly the same. Compared with the genome of Doper sheep, some regions may be missing in others, and some regions may be very different. Therefore, the genome of other sheep breeds may induce biases in variant calling, and SNPs and INDEL in those missing regions cannot be detected. That is to say, using the Doper sheep itself as a reference genome can greatly reduce the genetic differences caused by different breeds, thus, we can ensure the pertinence and accuracy of the SNP and INDEL that we found. It is for these reasons that the assembly of the Dorper sheep reference genome is necessary.

In the present work, we completed two tasks. First, a high-quality chromosome-level reference genome was assembled using Oxford Nanopore Technology (ONT) sequencing and chromatin conformation capture (Hi-C) technology. This research fills a gap in the reference genome of hair sheep. Second, we provided a pipeline for mining genetic information for composite breeds using allele-specific expression analysis. Using the assembled Dorper sheep genome as the reference genome, a data set of ancestor-specific differential SNP and INDEL loci was obtained using ancestral parent population genome re-sequencing data. Based on this dataset, Dorper sheep transcriptome data were used to verify allele specific expression sites. We then annotated these sites to identify the ASE genes. The study identified 21,289,550 SNPs in the ancestors, from which 5,701 ancestral unique SNP genotypes were obtained by genotyping. Similarly, the research detected 2,388,815 indels, which contained 476 ancestral genotypes. Based on outcomes from genotyping, 1,002 and 292 genes were identified from these SNPs and indels, respectively*.* These results contribute to our understanding of the genomic architecture of Dorper sheep.

## 2 Materials and Methods

### 2.1 Genome Assembly and Annotation

#### 2.1.1 Ethics Statement and Sample Collection

All animal experimental procedures in this study were approved by the Ethics Committee of the Lanzhou Institute of Husbandry and Pharmaceutical Sciences of the Chinese Academy of Agricultural Sciences. For whole-genome sequencing using the PromethION and Illumina sequencer instrument, blood sample of a Dorper sheep was collected from a healthy male from the Tianjin Aoqun Animal Husbandry Pty., Ltd. The blood was used for genomic DNA (gDNA) extraction, sequencing, and Hi-C library construction.

#### 2.1.2 DNA Extraction and Sequencing

High molecular weight genomic DNA was prepared from the blood using the SDS method followed by purification with QIAGEN® Genomic kit (Cat#13343, QIAGEN) according to the standard operating procedure provided by the manufacturer. The degradation and contamination of the extracted DNA were monitored on 1% agarose gel. DNA purity was then detected using a NanoDrop™ UV-Vis spectrophotometer (Thermo Fisher Scientific, United States), with an OD260/280 ranging from 1.8 to 2.0 and OD 260/230 between 2.0 and 2.2. DNA concentration was further measured using Qubit® 3.0 Fluorometer (Invitrogen, United States).

A total of 2 µg DNA per sample was used as input material for the ONT library preparation. After qualification, a size-selection of long DNA fragments was performed using the BluePippin system (Sage Science, United States). Next, the ends of DNA fragments were repaired, and A-ligation reaction was conducted using a NEBNext Ultra II End Repair/dA-Tailing Module (Cat# E7546, NEB). The adapter in the LSK109 kit (SQK-LSK109, Oxford Nanopore Technologies, United Kingdom) was used for further ligation reaction, and the Qubit® 3.0 Fluorometer was used to quantify the size of library fragments. Sequencing was then performed on a PromethION sequencer instrument (Oxford Nanopore Technologies, United Kingdom), using Nextomics.

#### 2.1.3 Data Quality Control

Base-calling was performed to convert the FAST5 files to fastq format with Guppy (V 3.2.2 + 9fe0a78) (https://github.com/nanoporetech/taiyaki). The raw reads of fastq format with a mean_qscore_template <7 were then filtered, resulting in pass reads.

#### 2.1.4 Genome Size and Heterozygosity Estimation

The k-mer analysis was performed using Illumina short-read data prior to genome assembly, to estimate genome size and heterozygosity. Briefly, quality-filtered reads were subjected to 17-mer frequency distribution analysis using the Jellyfish (v2.3.0) (Marçais & Kingsford, 2011). We estimated the genome size of the Dorper with the following equation: G = K-num/K-depth (where K-num is the total number of 17-mers, K-depth denotes the k-mer depth, and G represents the genome size). Further combination of the simulation data resulted from Arabidopsis with different heterozygosity and the frequency peak distribution of 17-mers was done to estimate the heterozygosity and repeat content of the Dorper genome.

#### 2.1.5 *De Novo* Assembly

For *de novo* genome assembly, ONT-only assembly was constructed using a string graph method with NextDenovo (v2.3.1) (https://github.com/Nextomics/NextDenovo.git). Considering the high error rate of ONT raw reads, the original subreads were first self-corrected using the NextCorrect module, resulting in consistent sequences (CNS reads). Comparison of the CNS reads was then performed with the NextGraph module to capture correlations of the CNS. Based on the correlation of CNS, the preliminary genome was assembled. To improve the accuracy of the assembly, the contigs were refined with Racon (v1.3.1) (https://github.com/isovic/racon.git) using ONT long reads and polished with Nextpolish (v1.3.0) (https://github.com/Nextomics/NextPolish.git) using Illumina short reads with default parameters. To discard possibly redundant contigs and generate a final assembly, similarity searches were performed using Redundans (Pryszcz & Gabaldón, 2016) with the parameters “-identity 0.9-overlap 0.9”.

The completeness of genome assembly was assessed using BUSCO (v4.0.5) (Simão, Waterhouse, Ioannidis, Kriventseva, & Zdobnov, 2015). To evaluate the accuracy of the assembly, all the Illumina paired-ended reads were mapped to the assembled genome using BWA (Burrows-Wheeler Aligner) (Li & Durbin, 2009) and the mapping rate, as well as genome coverage of sequencing reads, was assessed using SAMtools (v0.1.1855) (Kurtz et al., 2004). The base accuracy of the assembly was calculated with bcftools (v1.8.0) (http://samtools.github.io/bcftools/).

The coverage of expressed genes of the assembly was examined by aligning all the RNA-seq reads against the assembly using Hisat2 (v2.1.0) (http://ccb.jhu.edu/software/hisat/index.shtml) with default parameters. To avoid the inclusion of mitochondrial DNA sequences in the assembly, the draft genome assembly was submitted to the NT library to check contamination.

#### 2.1.6 Chromosome Assembly Based on Hi-C Technology

To anchor hybrid contigs onto the chromosome, genomic DNA was extracted from the Dorper male for the Hi-C library construction and sequencing *via* the Illumina Novaseq/MGI-2000 platform. In total, 370 million paired-end reads were generated from the libraries. Then, quality controlling of Hi-C raw data was performed using Hi-C-Pro (v2.8.1) (Servant et al., 2015). First, low-quality sequences (quality scores < 20), adaptor sequences, and sequences shorter than 30 bp were filtered out using fastp v0.19.8 (https://github.com/OpenGene/fastp), and then the clean paired-end reads were mapped to the draft assembled sequences using bowtie2 (v2.3.2) (http://bowtie-bio.sourceforge.net/bowtie2/index.shtml) to get the unique mapped paired-end reads. For further analysis, valid interaction paired reads were identified and retained by the HiC-Pro from uniquely mapped paired-end reads. Invalid read pairs, including dangling-end, self-cycle, re-ligation, and dumped products, were filtered by the HiC-Pro. The contigs were further clustered, ordered, and oriented onto chromosomes using LACHESIS (Burton et al., 2013). Finally, placement and orientation errors exhibiting obvious discrete chromatin interaction patterns were manually adjusted.

#### 2.1.7 Repetitive Sequence Detection

We first annotated tandem repeats using GMATA (v2.2) (https://sourceforge.net/projects/gmata/?source=navbar) and Tandem Repeats Finder (TRF) (v4.07b) (http://tandem.bu.edu/trf/trf.html) where GMATA identified simple repeat sequences (SSRs) and TRF recognized all tandem repeat elements in the whole genome. Transposable elements (TE) in the Dorper genome were then identified using a combination of ab initio and homology-based methods. Briefly, a ab initio repeat library for Dorper was first predicted using MITE-hunter (https://github.com/jburnette/MITE-Hunter) and Repeat Modeler (v open-1.0.11) (https://github.com/Dfam-consortium/RepeatModeler) with default parameters. The resultant library was then aligned to TEclass RepBase (http://www.girinst.org/repbase) to classify the type of each repeat family. For further identification of the repeats throughout the genome, RepeatMasker (Chen, 2004) was applied to search for known and novel TEs by mapping sequences against the *de novo* repeat library and RepBase (Bao, Kojima, & Kohany, 2015) TE library. Overlapping transposable elements belonging to the same repeat class were collated and combined.

#### 2.1.8 Gene Prediction and Annotation

Three independent approaches, including ab initio prediction, homology search, and reference guided transcriptome assembly, were used for gene prediction in a repeat-masked genome. GeMoMa (v1.6.1) (Keilwagen et al., 2016) was used to align the homologous peptides from related species to the assembly to obtain the gene structure information, which was the homolog prediction. For RNA-seq-based gene prediction, filtered mRNA-seq reads were aligned to the reference genome using STAR (vSTAR-2.7.3a) (https://github.com/alexdobin/STAR). The transcripts were then assembled using Stringtie (v1.3.4) (Pertea et al., 2015) and Open Reading Frames (ORFs) were predicted using TransDecoder (v2.0) (https://sourceforge.net/projects/transdecoder/) and PASA (v2.3.3) (Haas et al., 2003). For the *de novo* prediction, Augustus (v3.3.1) (Stanke & Waack, 2003), Genscan (v.3.1) (Burge & Karlin, 1997), GeneID (v.1.4) (Blanco, Parra, & Guigó, 2007), GlimmerHMM (v.1.2) (Majoros, Pertea, & Salzberg, 2004), GeneMarkS-T (v 4) (Besemer, Lomsadze, & Borodovsky, 2001), and SNAP (v.2006- 07-28) (Korf, 2004) were used with the default parameters. Finally, EVidenceModeler (v1.1.1) (Haas et al., 2008) was used to produce an integrated gene set from which genes with TEs were removed using the TransposonPSI package (http://transposonpsi.sourceforge.net/) and the miscoded genes were further filtered out.

Gene function information, motifs, and domains of the proteins were assigned by comparing them with public databases, including SwissProt, NR, KEGG, KOG, and Gene Ontology. The putative domains and GO terms of genes were identified using InterProScan (https://github.com/ebi-pf-team/interproscan/wiki) with default parameters. For the other four databases, BLASTP (v2.7.1) (https://blast.ncbi.nlm.nih.gov/Blast.cgi) was used to compare the Evidence Modeler-integrated protein sequences against the four well-known public protein databases with an E value cutoff of 1e−05; the results with the hit having the lowest E value were retained. Results from the five database searches were concatenated.

#### 2.1.9 Annotation of Non-Coding RNAs

To obtain the ncRNA (non-coding RNA), two strategies were used: searching against a database and prediction with a model. Transfer RNAs (tRNAs) were predicted using tRNAscan-SEM (v2.0) (Lowe & Eddy, 1997) with eukaryote parameters. MicroRNA, rRNA, small nuclear RNA, and small nucleolar RNA were detected using Infernal (v1.1.2) (http://eddylab.org/infernal/) to search the Rfam (Griffiths-Jones et al., 2005) database. The rRNAs and their subunits were predicted using RNAmmer (v1.2) (http://www.cbs.dtu.dk/services/RNAmmer/).

### 2.2 Ancestral Genomic Components Excavation

#### 2.2.1 Data Quality Control and Mapping

Genome resequencing data of ancestral breeds were downloaded from NCBI. The project numbers of three Persian sheep and 18 Dorset sheep were PRJEB39179 and PRJNA675420, respectively. The RNA-Seq data of six Dorper sheep were downloaded from NCBI BioProjects PRJNA631066.

Before mapping to Dorper reference genome, the data were processed to filter out low-quality reads. The Fastp was used to filter the original data with default parameters. The filtering conditions were as follows: 1) removing adapters of reads; 2) removing reads containing more than 10% of unknown nucleotides; 3) removing low-quality reads containing more than 50% of low-quality (Q-value ≤10) bases; and 4) removing reads for which the average base quality value was less than 20. The filtered reads were then mapping to the Dorper sheep reference genome with BWA v0.19.8 (https://github.com/lh3/bwa).

#### 2.2.2 Variants Detection and Annotation

SNPs and indels were called and filtered using GATK v4.0 (https://gatk.broadinstitute.org/hc/en-us) and VCFtools v0.1.13 (http://vcftools.sourceforge.net/). First, GATK quality value and density were used for filtering. SNPs were filtered with the following parameters: --filter Expression “QUAL < 30.0 || QD < 2.0 || FS > 60.0 || MQ < 40.0 || SOR > 4.0” -cluster 3 -window 10. The indels were filtered with the following parameters: --filter Expression QUAL < 30.0 || QD < 2.0 || FS > 200.0 || SOR > 10.0 || MQ < 40.0. Then VCFtools was used to filter loci by allele frequency and depth with the following parameters: --min-alleles 2 --max-alleles 2 --min-meanDP 5 --maf 0.05 --max-missing 0.5. ANNOVAR (https://annovar.openbioinformatics.org/en/latest/) was used for the annotation of SNPs and indels.

#### 2.2.3 Allele-Specific Expression Analysis

Allele counts at SNP and indel positions were retrieved using an in-house Python script. The screening conditions for specific SNPs and indels in ancestors (Dorset and Persian sheep) were as follows: 1) genotypes of ancestors were confirmed based on the proportion of the major genotype of ancestors in the population being > 75% (e.g., among 18 Dorset sheep, when a genotype appeared in more than 14 individuals, it can be considered as a major genotype) and 2) to establish the ancestral source of an offspring allele, only the homozygous major genotypes in inconsistent sites were retained.

SNP and indel genotyping and screening procedures of Dorper sheep were as follows: 1) considering the most commonly missing sites in the intergenic region of the transcriptome data compared to the re-sequencing data, the sites with >50% missing data were filtered out; 2) the major genotype proportion of Dorper sheep must be >60% (e.g., if six genotypes were detected, when a genotype appeared in more than four individuals, it could be considered as a major genotype at this locus). Subsequently, heterozygous and missing genotypes were filtered out; 3) by comparing the major genotypes of the Dorper sheep with the main genotypes of the ancestors (Dorset and Persian sheep), the ancestor from which the site was mainly derived from can be determined; 4) through the variant annotation information which was obtained in the previous step, the information for each gene corresponding to the variation site was confirmed. This information included which allele and gene structure a variant is located on; and 5) Finally, we counted the number of SNPs and indels for each allele to determine which ancestor the allele is mainly derived from.

## 3 Results

### 3.1 Genome Assembly and Annotation

#### 3.1.1 Genome Assembly

**FIGURE 1:**
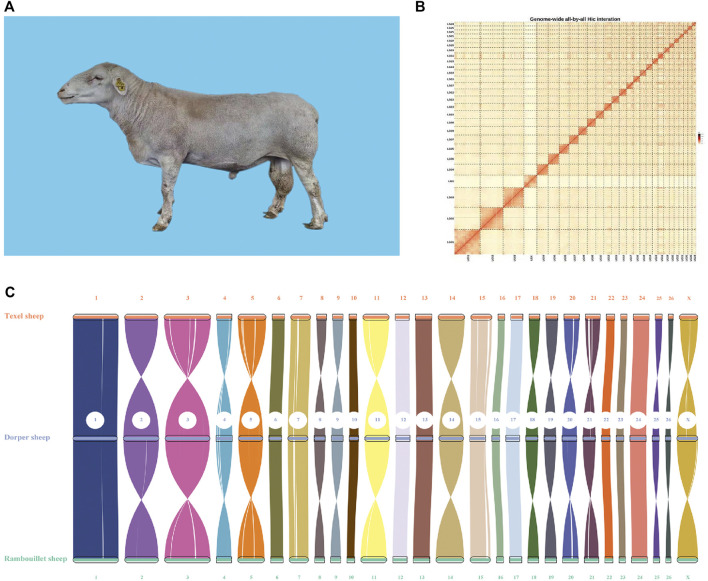
Image and genome quality of Dorper sheep. **(A)** Image of Dorper Sheep. **(B)** Genome-wide all-by-all Hi–C interaction. **(C)** Syntenic analysis of assembled genome.

In this study, ∼163.08 Gb of filtered Illumina short-read sequencing data were obtained from the Dorper sheep (Figure 1A; Supplementary Table S1). The size of the Dorper genome was estimated to be around 2.65 Gb with 0.4% heterozygosity (Supplementary Figure S1).

After filtering out adaptor sequences, ∼224.57 Gb of ONT subreads with an average length of 21.17 Kb was obtained. These clean reads were used to *de novo* assemble the genome which was refined and polished with Illumina short reads. About 91.58% of the conserved genes could be detected in the Dorper genome by the BUSCO software using mammalia_odb10 dataset, confirming the high completeness of the obtained genome (Supplementary Table S2).

Finally, we used the Hi-C technique to anchor assembly contigs in 27 chromosomes (2n = 54). We found that 831,180,895 uniquely mapped paired-end reads were generated and occupied ∼70.41% of the total clean paired-end reads (1,180,468,259). The frequency of contig interactions was estimated on the basis of pairs mapped to the contigs. We found that 107 contigs, representing 77.54% of all contigs and 99.57% of the whole genome nucleotide bases were successfully anchored on 27 chromosomes (Supplementary Table S3 and Figure 1B). The final assembly resulted in a 2.64 Gb genome with a contig N50 of 73.33 Mb. The genome consisted of 140 contigs, with the longest contig being 158.3 Mb (Table 1).

**TABLE 1:** Genome assembly statistics of Dorper sheep.

Statistic	Contig length (bp)	Contig number
N50	73,326,320	13
N50	64,997,665	17
N50	43,243,940	21
N50	37,997,576	28
N50	22,706,521	37
Longest	158,282,255	1
Total	2,648,309,365	140
Length >= 1 kb	2,648,309,365	140
Length >= 2 kb	2,648,309,365	140
Length >= 5 kb	2,648,309,365	140

#### 3.1.2 Genome Characteristics

**FIGURE 2:**
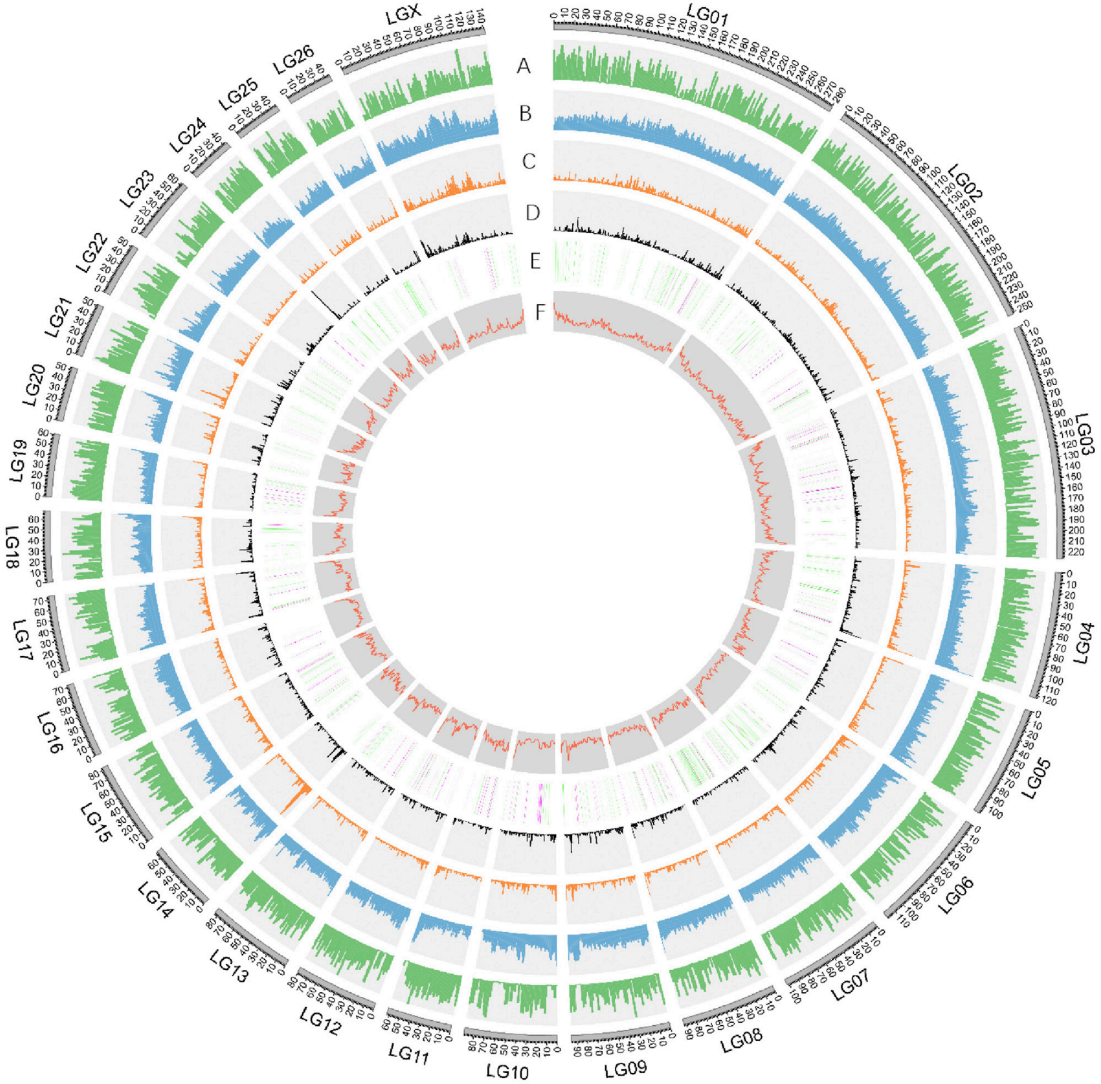
Characterization of Dorper sheep genome. Circos diagram showing (outer to inner): A. Gene density (genes per 1 Mb window). B. Repeat density (repetitive sequence per in 1 Mb window). C. Gypsy-type LTR density. D. Copia-type LTR density. E. The numbers and distribution of ASE genes derived from DSS and PSS were represented in green and purple respectively. F. GC content in sliding windows of 1 Mb across each chromosome.

We found that the GC content of the Dorper genome was 41.99% (Figure 2, Supplementary Figure S2), which was similar to that of other domestic sheep breeds (42.12%), snow sheep (42.12%), and goats (41.5%) (Upadhyay et al., 2020). TEs contributed 1,202,782,366 bp of the genome and accounted for 45.42% of the genome length (Supplementary Table S4). We found that class I TEs (RNA transposons or retrotransposons) occupied 42.54% of the genome. The most abundant retrotransposons found in the Dorper genome were long interspersed nuclear elements (LINE), which constituted 78.51% of all identified class I transposons. Moreover, the Dorper genome was not rich in class II TEs (DNA transposons), which occupied only 2.68% of the genome content. The assembly quality statistics comparison is listed in Table 2.

**TABLE 2:** Assembly quality statistics comparison.

Assembly statistic	ASM1914517V1	ARS-UI_Ramb_v2.0	Oar_rambouillet_v1.0	Oar_v4.0
Total Length (Mb)	2,648.31	2,628.15	2,869.91	2,615.52
Contig No.	140	226	7,486	48,482
Contig N50 (bp)	73326320	43,178,051	2,850,956	145,655
Contig L50 (No. of contigs)	13	24	263	5,206
Complete, single-copy BUSCOs (%)	91.58	93.9	93.0	91.2
Complete, duplicated BUSCOs (%)	1.6	2.1	2.6	1.6
Percent of fragmented BUSCOs	2.22	0.9	1.1	2.4
Percent of missing BUSCOs	6.2	3.1	3.3	4.8

The obtained consensus gene set included 20,450 protein-coding genes (Supplementary Table S5). For the completeness of protein-coding genes, 94.18 and 2.22% of the “total complete BUSCOs” and “fragmented BUSCOs” were identified by BUSCO annotation, respectively. The average coding sequence length (CDS), average exon length, and average intron length were 1,571, 160, and 5,477 bp, respectively (Supplementary Figure S5 and Supplementary Table S6). There were on average 9.8 exons per gene. We found functional annotation for 18,491 protein-coding genes, which represented about 90.42% of all the genes (Supplementary Table S7). Non-protein-coding genes included 251,525 tRNAs, 412 rRNAs, and 769 microRNAs (miRNAs) (Figure 2, Supplementary Table S8).

To evaluate quality of the genome annotation, the collinearity analysis of the Dorper sheep was conducted with Texel (Oar_v4.0) and Rambouillet (ARS-UI_Ramb_v2.0) sheep. The high collinearity observed among these three genomes illustrated that the accuracy of the Dorper genome assembly and annotation was high (Figure 1C).

### 3.2 Ancestral Genomic Components

#### 3.2.1 Genomic Variants

A single-nucleotide polymorphism (SNP) and an indel database were developed between Dorset sheep (DSS), Persian sheep (PSS), and Dorper sheep (DPS). The re-sequencing data of DSS and PSS achieved an average depth of 8× and a mapping rate of 99.70% (Supplementary Table S9). The RNA-Seq data of DPS achieved an average depth of 4× and a mapping rate of 99.74% (Supplementary Table S9). A total of 21,289,550 SNPs were found in all data. The proportion of transitions (15,147,780, 71.15%) was much higher than that of transversions (6,141,770, 28.85%). The transition: transversion ratio was 2.47, which was similar to that found in other studies (Guan et al., 2016). A total of 2,388,815 indels were sought out among the three breeds. There were more deletions (1,306,121) than insertions (1,082,694).

#### 3.2.2 Allele-Specific Expression Analysis

The ancestral alleles for all the SNPs and indels were inferred by comparing these variants to the Dorper sheep genome (Table 3, Table 4, Figure 2). Initially, we determined that 5,701 SNPs (1,000 bp centered around the gene site) were located inside 1,002 genes with at least one discriminating SNP, of which 260 SNP alleles from the Persian sheep, 723 SNP alleles from the Dorset sheep, and 19 SNP alleles were present in both breeds (Supplementary Table S10). At the above 5,701 SNP sites, 1,247 SNPs were from Persian sheep and 4,454 SNPs were from Dorset sheep. The same method for indels was used. The analysis detected 456 indels located inside 294 genes. In these indel mutant alleles, 66 alleles belonged to Persian sheep and 228 alleles to Dorset sheep (Supplementary Table S11).

**TABLE 3:** The top ten genes from Persian sheep

	Gene symbol	Full name	CHR
1	*SOCS2*	Gene—Suppressor Of Cytokine Signaling 2	3
2	*MYCB2*	MYC Binding Protein 2	11
3	*ARFGEF2*	ADP Ribosylation Factor Guanine Nucleotide Exchange Factor 2	12
4	*SEC31*	SEC31 Homolog A, COPII Coat Complex Component	6
5	*EXT2*	Exostosin Glycosyltransferase 2	13
6	*ITPR1*	Inositol 1,4,5-Trisphosphate Receptor Type 1	21
7	*ARIH1*	Ariadne RBR E3 Ubiquitin Protein Ligase 1	8
8	*LRIG1*	Leucine Rich Repeats And Immunoglobulin Like Domains 1	21
9	*UCHL4*	Ubiquitin C-Terminal Hydrolase L4	11
10	*UBP22*	UBIQUITIN-SPECIFIC PROTEASE 22	20

**TABLE 4:** The top ten genes from Dorset sheep.

	Gene symbol	Full name	CHR
1	*UB2E2*	Ubiquitin Conjugating Enzyme E2 E2	26
2	*KCNQ5*	Potassium Voltage-Gated Channel Subfamily Q Member 5	9
3	*PCNX1*	Pecanex 1	8
4	*ARHGAP24*	Rho GTPase Activating Protein 24	6
5	*STX8*	Syntaxin 8	20
6	*KIAA0586*	KIAA0586	8
7	*MRPL42*	Mitochondrial Ribosomal Protein L42	3
8	*KTN1*	Kinectin 1	8
9	*DCAF5*	DDB1 And CUL4 Associated Factor 5	8
10	*UTRN*	Utrophin	10

#### 3.2.3 Enrichment Analysis of ASE Genes

To explore the role of the genes carrying ASE, functional enrichment analyses were performed. Gene Ontology (GO) enrichment analysis of the 260 SNP alleles of Persian sheep showed that there were seven significant GO terms in molecular function (MF) and two significant GO terms in biological process (BP) (*p* < 0.05) (Supplementary Table S12, Supplementary Figure S4). GO enrichment analysis of the 723 SNP alleles of Dorset sheep showed that GO terms were significantly enriched in 125 ASE genes (*p* < 0.05), which were mainly involved in eight MF, five BP, and one Cellular Component (CC) (Supplementary Table S13, Supplementary Figure S5). For indel mutant alleles, GO enrichment analysis identified eight significantly (*p* < 0.05) enriched GO terms composed of four GO terms in MF and four GO terms in BP in Persian sheep (Supplementary Table S14, Supplementary Figure S6). The GO enrichment analysis showed that the indel mutant alleles of Dorset sheep were involved in nine GO terms (*p* < 0.05): four GO terms in MF, four GO terms in MF, and one GO terms in CC (Supplementary Table S15, Supplementary Figure S7).

KEGG analysis resulted in 61 significant (*p* < 0.05) SNP alleles of Persian sheep to be annotated to 15 KEGG pathways (Supplementary Table S16, Supplementary Figure S8). According to the annotation of KEGG, 218 SNP alleles of Dorset sheep were significantly (*p* < 0.05) annotated to 27 KEGG pathways (Supplementary Table S17, Supplementary Figure S9). After KEGG analysis, indel alleles of Persian sheep were mapped to seven significant KEGG pathways (*p* < 0.05) (Supplementary Table S18, Supplementary Figure S10). Indel alleles of Dorset sheep were mapped to 11 significant KEGG pathways (*p* < 0.05) (Supplementary Table S19, Supplementary Figure S11).

## 4 Discussion

### 4.1 Genome Assembly and Annotation

Returning to the question posed at the beginning of this study, it is now possible to state that the first chromosome-scale reference genome of hair sheep is assembled. As of 2022, more than 15 domestic sheep breeds genome sequences have been recorded in the National Center for Biotechnology Information (NCBI). From the first version of the sheep reference genome (PRJNA33937) published by The International Sheep Genomics Consortium in 2010 to the 13 versions of the sheep genome involved in the pan-genome article published by Li et al., in 2021 (Li et al., 2021), sheep genome sequencing assembly has undergone a process from first-generation sequencing technology to third-generation sequencing technology. The ARS-UI_Ramb_v2.0 (Davenport et al., 2022), and previously Oar_rambouillet_v1.0, and Oar_v4.0 (Jiang et al., 2014) is the current reference genome for sheep. Comparing these versions, we found that the Contig N50 of Oar_v4.0 to ARS-UI_Ramb_v2.0 became longer gradually, from 145 kb to 43 M, indicating an obvious improvement in the assembly level. The advent of third-generation sequencing, long read-sequencing has meant scientists can now generate many sheep genomes from different breeds and populations from around the world. Recently a sheep pan-genome was published (Li et al., 2021) that included long-read genome assemblies 13 breeds. Many years of natural and artificial selection have produced abundant phenotypic variation in sheep populations. Different breeds contribute genetic diversity to global sheep genetic resources. Genome assembly of different breeds helps to reveal the origins and evolutionary forces of sheep population structure and constitutes a valuable resource for sheep breeding programs and genetic diversity studies.

Like most genome assembly strategies nowadays, third-generation sequencing technology was used in this study. The major advantage of the third-generation sequencing technology is the long read length. Specifically, we used Oxford Nanopore Technology (ONT) sequencing strategy which was also used in Rambouillet (Davenport et al., 2022) and Hu (Li et at., 2021) sheep. As a result, the final assembled genome size was 2.64 Gb with a scaffold N50 of 101.9 Mb and contig N50 of 73 M. The size of this genome is within the range of published sheep genome sizes, ranging from 2.61 Gb for the Texel sheep (Jiang et al., 2014) to 2.90 Gb for the East Friesian sheep (PRJNA721520). Compared to the other sheep breeds, the scaffold N50 of the Dorper sheep assembly is in the top quartile. In sheep genomes assembled using PacBio and ONT, the longest scaffold N50 is 107.7 M for Rambouillet sheep (Davenport et al., 2022) and the shortest one is 100 M for Texel sheep (Jiang et al., 2014). The contig N50 in our study is longer than those of most sheep genome assemblies. Comparison of Contig N50 with reference genomes is also detailed in Table 2 of the manuscript. Our study implied that more and more high-quality sheep genomes of different breeds will be assembled with advances in sequencing technologies and assembly methods and reduced sequencing costs. As the study in Li et al., 13 sheep breeds genomes were assembled at the same time, which include Dorper sheep (Li et at., 2021). The Dorper sheep genome published in the study of Li et al. was assembled using PacBio HiFi sequencing. There were certain unique points to their study, compared with ours. Especially, they assembled 2 haplotype-resolved genome assemblies based on HIFI data. However, their assembly level is still in scaffolds, we generated Hi-C data from the same individual to cluster, order, and orient contigs onto chromosomes. We provided a new assembly for Dorper and a detailed description in this manuscript including annotation and analysis of ASE, providing additional resources for the Dorper breed to those included in the pangenome created by Li et al.

### 4.2 Ancestral Genomic Components

**FIGURE 3:**
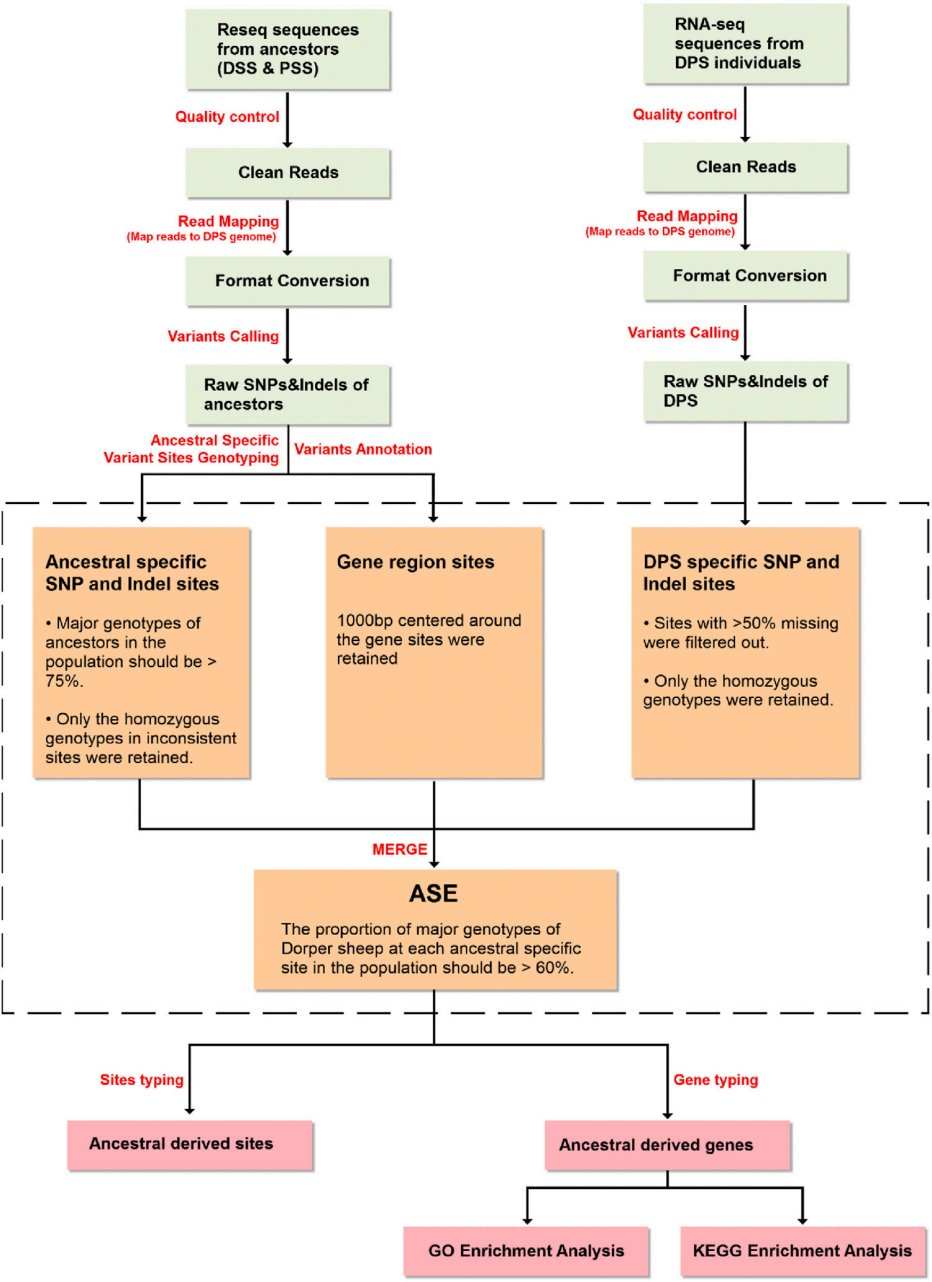
Bioinformatics pipeline for allelic genes expression estimation.

Allele-specific expression (ASE) analysis identified multiple ASE SNPs and ASE indels in Dorper sheep which were derived from ancestors (Figure 3). These ASE genes are related to many essential traits, including growth (*IGF1, DAAM, PHF17, SYNE2, OST1, KIF20*), immune responses (*ABCC4, ARI1, CELF2, TMCO3*), and reproduction (*TAF4B, HTF4, STK10, LAYN*). Here, we had some interesting findings from the enrichment analyses of these alleles. Several GO terms were found in both ancestors. For instance, metal ion binding, protein phosphorylation, protein serine/threonine kinase activity, regulation of Rho protein signal transduction, and Rho guanyl-nucleotide exchange factor activity. Protein phosphorylation is an important factor in the transition from muscle to edible meat (Huang, Larsen, & Lametsch, 2012). It also has important effects on many physiological and biochemical reactions in muscles. Rho is active when bound to GTP and inactive when bound to GDP. It is also known to participate in many physiological activities including cell migration, adhesion, cytokinesis, proliferation, differentiation, and apoptosis, and to a greater extend cell transformation (Heasman & Ridley, 2008). Among KEGG pathways, ubiquitin-mediated proteolysis, autophagy, lysosome, the mTOR signaling pathway, and cellular senescence were detected in both ancestral breeds. The common GO terms and KEGG pathways in these ancestors indicate that growth and development related traits of Dorper sheep are a result of combinations of the maternal and paternal ancestral genomes.

For Persian sheep, there were specific GO terms involved in lipid-related processes, such as lipid binding and galactosyltransferase activity. Galactosyltransferase activity is a catalysis of the transfer of a galactosyl group to an acceptor molecule, typically another carbohydrate or a lipid (Jensen, Schultink, Keegstra, Wilkerson, & Pauly, 2012). These results are consistent with characteristics of Persian sheep, which is a fat-tailed breed (Lundie, 2011). Therefore, we suggest that Persian sheep made more contributions to traits related to fat deposition. In Persian sheep, the metabolic pathways mainly involve carbohydrate metabolisms, such as fructose and mannose metabolism, glycosphingolipid biosynthesis, and other types of O−glycan biosynthesis. The Persian sheep originated in the arid regions of east Africa in what is now Somalia. Their glycolysis pathway and catabolism of carbohydrates were enhanced under drought conditions (Bowne et al., 2012). These metabolism-related genes may explain the genetic basis of drought resistance in Dorper sheep. The GO terms from Dorset sheep were typically involved in muscle-associated events. Such as myosin complex and motor activity. Myosin is a superfamily of motor proteins associated with muscle contraction and a wide range of other motility processes in eukaryotes (Sellers, 2000). In Dorset sheep, the related organismal systems pathways mainly involve multiple signaling pathways. According to these results, we can infer that Dorset sheep have more impact than Persian sheep in the growth rate, carcass quality, and carcass yield of Dorper sheep.

This study set out to gain a better understanding of a hair sheep genome and the genetic basis of Dorper sheep. The present results are significant in at least two major respects. First, we provide the first high-quality reference genome of hair sheep, representing a valuable resource for sheep genetic studies. Second, the evidence from this study suggests that the pipeline we constructed for heterosis evaluation based on ASE genes detection is feasible. Through this approach, we found a number of ASE genes in the ancestral population that potentially contributed to the genetic mechanism of important economic traits of Dorper sheep. The method that we designed to reveal the heterosis might help others to evaluate composite breeds, which has important implications for crossbreeding and improvement through the breeding and selection of new high-quality cultivar sheep. Despite these promising results, questions remain. The adaptation and phenotypic differences of the Dorper sheep may be mediated by a complex network of genes that act in tandem, rather than by the action of a single candidate gene (Lv et al., 2014; Kim et al., 2015). It is therefore difficult to directly draw conclusions regarding the genetic mechanisms underlying the observed traits based only on ASE. Furthermore, with only six Dorper, three Persian, and 18 Dorset sheep data set, the sample size was probably too small to obtain reliable estimates. Further studies are required to better understand the mechanisms underlying the genome of Dorper sheep. Notwithstanding the relatively limited sample, this work offers valuable insights into genetic basis research for composite breeds.

## Data Availability

The datasets presented in this study can be found in online repositories. The names of the repository/repositories and accession number(s) can be found below: https://www.ncbi.nlm.nih.gov/, PRJNA721526.
