# Supplementary material for: Genetic Basis of Dorper Sheep (Ovis aries) Revealed by Long-Read De Novo Genome Assembly
Source: Front Genet. 2022 Apr 11;13:846449. doi: 10.3389/fgene.2022.846449 (PMC9035736; doi:10.3389/fgene.2022.846449)
Supplement: Supplementary file 1 [file DataSheet1.ZIP › Supplementary_material/(tableS1-S9,Figure S1-S11)Suppinfo.doc]

Supplementary Material

**Tabel S1. Genome assembly statistics of Dorper**

| Sequencing libraries | Total reads | Total bases(Gb) | Clean reads | Clean bases(Gb) |
| --- | --- | --- | --- | --- |
| Illumina reads | 1,182,362,424.00 | 177.00 | 1,171,090,658.00 | 163.00 |
| Ont reads | 10,605,515.00 | 224.57 | - | - |
| Hi-C reads | 2,384,560,974.00 | - | 2,360,936,518.00 | 353.00 |

**Table S2. Summary of BUSCO analysis results**

| Type | Number | Percent(%) |
| --- | --- | --- |
| Complete BUSCOs (C) | 8,449 | 91.58 |
| Complete and single-copy BUSCOs (S) | 8,301 | 89.97 |
| Complete and duplicated BUSCOs (D) | 148 | 1.60 |
| Fragmented BUSCOs (F) | 205 | 2.22 |
| Missing BUSCOs (M) | 572 | 6.20 |
| Total BUSCO groups searched | 9,226 | 100.00 |

**Table S3. Statistics of the assembly contigs anchored in 27 chromosomes of Dorper**

| Chr | Size(bp) | Number of contigs |
| --- | --- | --- |
| LG01 | 280,961,601 | 10 |
| LG02 | 251,724,035 | 2 |
| LG03 | 227,474,520 | 6 |
| LG04 | 122,323,029 | 4 |
| LG05 | 119,201,786 | 2 |
| LG06 | 108,993,333 | 1 |
| LG07 | 101,972,938 | 7 |
| LG08 | 95,575,060 | 1 |
| LG09 | 92,267,707 | 1 |
| LG10 | 89,574,757 | 4 |
| LG11 | 83,897,696 | 4 |
| LG12 | 82,316,136 | 7 |
| LG13 | 80,583,108 | 5 |
| LG14 | 73,631,867 | 2 |
| LG15 | 72,267,558 | 2 |
| LG16 | 68,537,076 | 5 |
| LG17 | 66,909,457 | 2 |
| LG18 | 62,846,695 | 1 |
| LG19 | 62,792,272 | 3 |
| LG20 | 60,700,000 | 1 |
| LG21 | 52,389,156 | 2 |
| LG22 | 51,860,030 | 2 |
| LG23 | 51,530,779 | 3 |
| LG24 | 45,738,923 | 6 |
| LG25 | 45,074,839 | 3 |
| LG26 | 43,926,931 | 4 |
| LGX | 141,720,241 | 17 |
| Total | 2,636,791,530 | 107 |

**Table S4 . Characteristics of TEs identified in Dorper**

| Class | Order | Super family | Number  of elements | Length  of sequence (bp) | Percentage  Of sequence (%) |
| --- | --- | --- | --- | --- | --- |
| ClassI |  |  | 4027381 | 1126623228 | 42.54 |
|  | LINE |  | 2679306 | 884601976 | 33.4 |
|  |  | Unknown | 288161 | 127674866 | 4.82 |
|  |  | RTE-BovB | 1198875 | 382065135 | 14.43 |
|  |  | L1 | 958979 | 339409252 | 12.82 |
|  |  | L2 | 172918 | 29624571 | 1.12 |
|  |  | CR1 | 19496 | 3079867 | 0.12 |
|  |  | Other | 40877 | 2748285 | 0.1 |
|  | LTR |  | 862497 | 180309205 | 6.81 |
|  |  | ERV1 | 193103 | 44145324 | 1.67 |
|  |  | ERVK | 38308 | 10008904 | 0.38 |
|  |  | ERVL-MaLR | 158684 | 37327896 | 1.41 |
|  |  | Gypsy | 300066 | 45376680 | 1.71 |
|  |  | Unknown | 49131 | 14693936 | 0.55 |
|  |  | ERVL | 107599 | 28140623 | 1.06 |
|  |  | Other | 15606 | 615842 | 0.02 |
|  | SINE |  | 485578 | 61712047 | 2.33 |
|  |  | Unknown | 178577 | 24707042 | 0.93 |
|  |  | MIR | 252829 | 31105388 | 1.17 |
|  |  | tRNA-Core-RTE | 40890 | 5057660 | 0.19 |
|  |  | Other | 13282 | 841957 | 0.03 |
| Class | II |  | 654815 | 76159138 | 2.88 |
|  |  | DNA | 625620 | 71068747 | 2.68 |
|  |  | Unknown | 71231 | 4633876 | 0.17 |
|  |  | TcMar-Mariner | 8684 | 2828050 | 0.11 |
|  |  | TcMar-Tigger | 62774 | 13713709 | 0.52 |
|  |  | hAT-Charlie | 177890 | 28944992 | 1.09 |
|  |  | hAT-Tip100 | 47509 | 7039937 | 0.27 |
|  |  | Other | 257532 | 13908183 | 0.53 |
|  | MITE |  | 10816 | 4106556 | 0.16 |
|  |  | Unknown | 10816 | 4106556 | 0.16 |
|  | RC |  | 18379 | 983835 | 0.04 |
|  |  | Other | 18379 | 983835 | 0.04 |
| Total | TEs |  | 4682196 | 1202782366 | 45.42 |

**Table S5. Gene annotation statistics of the Dorper**

| Gene set | Total number of genes | Average gene length(bp) | Average  CDS  length(bp) | Average exons number per gene | Average exon length(bp) | Average intron length(bp) |
| --- | --- | --- | --- | --- | --- | --- |
| De novo | 23,868 | 40,999.11 | 1,521.39 | 9.07 | 167.81 | 4,894.32 |
| Homology | 19,731 | 37,822.07 | 1,547.99 | 8.48 | 182.63 | 4,851.94 |
| RNA-seq | 10,828 | 42,138.93 | 2,774.70 | 16.79 | 165.26 | 2,493.05 |
| Final set | 20,450 | 49,741.48 | 1,571.31 | 9.8 | 160.41 | 5,476.77 |

| Species | Total number of genes | Average transcript length(bp) | Average CDS length(bp) | Average exons number per gene | Average exon length(bp) | Average intron length(bp) |
| --- | --- | --- | --- | --- | --- | --- |
| Dorper sheep | 20,450 | 49,741.48 | 1,571.31 | 9.8 | 160.41 | 5,476.77 |
| *Bos_taurus* | 20,699 | 44,674.19 | 1,636.76 | 9.3 | 176.04 | 5,186.82 |
| *Capra_hircus* | 20,151 | 44,659.05 | 1,663.06 | 9.47 | 175.6 | 5,075.88 |
| *Homo_sapiens* | 22,517 | 32,568.93 | 1,457.05 | 8.25 | 176.65 | 4,292.30 |
| *Mus_musculus* | 22,139 | 37,821.80 | 1,581.18 | 8.75 | 180.64 | 4,674.31 |
| *Ovis_aries* | 20,715 | 44,055.05 | 1,643.32 | 9.39 | 174.98 | 5,054.10 |
| *Ovis_Orientalis* | 20,042 | 47,085.44 | 1,640.85 | 9.61 | 170.72 | 5,277.25 |

**Table S6. Characteristics of genes identified in the Dorper**

**Table S7. Functional annotation of predicted genes of Dorper**

| Type | | Number | Percent (%) |
| --- | --- | --- | --- |
| Annotation | Swissprot | 18,143 | 88.72 |
|  | KEGG | 12,864 | 62.9 |
|  | KOG | 12,866 | 62.91 |
|  | GO | 12,813 | 62.66 |
|  | NR | 18,376 | 89.86 |
| Total | Annotated | 18,491 | 90.42 |
|  | Gene | 20,450 | - |

**Table S8. Summary of non-protein-coding genes in the Dorper**

| Type | | Copy Number | Average Length(bp) | Total Length(bp) |
| --- | --- | --- | --- | --- |
| rRNA (412) | 18S | 3 | 1,802.67 | 5,408 |
|  | 28S | 7 | 5,856.29 | 40,994 |
|  | 5.8S | 12 | 151.25 | 1,815 |
|  | 5S | 390 | 111.93 | 43,651 |
| sRNA (3,079) | snRNA | 685 | 115.27 | 78,959 |
|  | miRNA | 769 | 79.44 | 61,089 |
|  | spliceosomal | 1,256 | 114.54 | 143,862 |
|  | other | 369 | 190.06 | 70,133 |
| Regulatory | cis-regulatory elements | 552 | 66.7 | 36,816 |

| **Table S9. Read Mapping** | | | | | | |
| --- | --- | --- | --- | --- | --- | --- |
| #Sample | TotalReads | MapRate | UniqMapRate | Covrage | Depth | CovrageBase |
| AST49 | 122,756,358 | 99.44% | 96.67% | 98.59 | 5.6 | 2,599,586,458 |
| AST48 | 185,009,218 | 99.81% | 97.37% | 99.27 | 8.36 | 2,617,592,442 |
| AST45 | 186,845,658 | 99.82% | 97.12% | 99.3 | 8.34 | 2,618,422,636 |
| AST44 | 185,938,980 | 99.77% | 97.09% | 99.29 | 8.31 | 2,618,108,583 |
| AST47 | 161,223,042 | 99.73% | 96.90% | 99.09 | 7.35 | 2,612,845,395 |
| AST46 | 161,328,490 | 99.86% | 97.16% | 99.07 | 7.18 | 2,612,357,626 |
| AST43 | 130,073,167 | 99.79% | 97.56% | 98.71 | 5.94 | 2,602,703,921 |
| AST42 | 111,019,497 | 99.76% | 97.37% | 97.85 | 5.02 | 2,580,151,287 |
| AST12 | 160,527,823 | 99.88% | 96.87% | 98.55 | 5.94 | 2,598,659,264 |
| BSY45 | 315,073,115 | 99.65% | 98.37% | 98.03 | 17.58 | 2,584,763,049 |
| BSY44 | 248,554,625 | 99.91% | 98.66% | 99.53 | 13.39 | 2,624,377,490 |
| BSY43 | 286,249,172 | 99.87% | 98.51% | 99.59 | 15.68 | 2,625,873,282 |
| AST19 | 177,946,123 | 99.87% | 96.81% | 98.73 | 6.49 | 2,603,195,736 |
| AST56 | 164,685,609 | 99.57% | 94.96% | 98.78 | 7.45 | 2,604,652,086 |
| AST57 | 141,342,987 | 99.60% | 92.11% | 98.64 | 6.3 | 2,601,003,237 |
| AST54 | 140,729,173 | 99.39% | 95.52% | 98.44 | 6.4 | 2,595,646,053 |
| AST55 | 134,875,471 | 99.52% | 96.77% | 98.45 | 6.08 | 2,596,051,313 |
| AST52 | 208,767,416 | 99.45% | 96.35% | 99.35 | 9.34 | 2,619,633,682 |
| AST53 | 111,548,458 | 99.48% | 95.29% | 97.6 | 5.05 | 2,573,612,384 |
| AST50 | 160,693,502 | 99.72% | 96.27% | 99.04 | 7.21 | 2,611,558,697 |
| AST51 | 173,904,146 | 99.82% | 96.82% | 99.2 | 7.76 | 2,615,618,970 |
| DBY39 | 114,165,288 | 99.77% | 81.79% | 16.38 | 4.37 | 431,950,450 |
| DBY42 | 98,327,684 | 99.75% | 83.43% | 11.67 | 3.69 | 307,665,739 |
| DBY43 | 105,404,277 | 99.78% | 82.20% | 9.87 | 3.79 | 260,210,514 |
| DBY40 | 123,097,363 | 99.78% | 82.90% | 14.15 | 4.53 | 372,981,206 |
| DBY41 | 114,940,508 | 99.64% | 84.36% | 9.52 | 4.24 | 250,920,671 |
| DBY44 | 97,638,868 | 99.74% | 84.52% | 8.57 | 3.48 | 226,103,304 |

**Figure S1. Sample k-mer distribution curve and heterozygosity simulation curve.**

**Figure S2. The GC depth distribution of the Dorper genome**

**Figure S3. Comparison of gene structure characteristics in Dorper to those of other members**.

**Figure S4. Top 20 GO terms of the 260 SNP alleles of Persian sheep**

**Figure S5. Top 20 GO terms of the 723 SNP alleles of Dorset sheep**

**Figure S6. Top 20 GO terms of the indel alleles of Persian sheep**

**Figure S7. Top 20 GO terms of the indel alleles of Dorset sheep**

**Figure S8. Top 20 KEGG pathways of the SNP alleles of Persian sheep**

**Figure S9. Top 20 KEGG pathways of the SNP alleles of Dorset sheep**

**Figure S10. Top 20 KEGG pathways of the indel alleles of Persian sheep**

**Figure S11. Top 20 KEGG pathways of the indel alleles of Dorset sheep**
